# Metrotomy

**Published:** 1882-04

**Authors:** J. V. Newton


### VI.—METROTOMY.

REPORTED BY J. V. NEWTON, V.S.

I was called, some weeks ago, to see a cow near this city who, the night before I arrived, had been, to all appearance, perfect health. The morning prior to my visit she had given birth to a calf, and the uterus had become inverted. When I arrived at the farm and made an examination, I found the lower portion of the uterus badly lacerated, and that it was rapidly falling into a gangrenous condition. Under such circumstances the only thing to be done was amputation of the same.

I placed a strong ligature well above the diseased portion, drawing it very tight to prevent, if possible, all hemorrhage. I then severed the organ one inch posterior to the ligature. Practically there was no hemorrhage attending the operation. For a few days I had the cow fed on a spare and laxative diet. The recovery was perfect, and in two weeks she was feeding two calves.

TOLEDO, OHIO, February, 1882

